# Supplementary material for: Evaluating and mitigating clinical samples matrix effects on TX-TL cell-free performance
Source: Sci Rep. 2022 Aug 12;12:13785. doi: 10.1038/s41598-022-17583-4 (PMC9374283; doi:10.1038/s41598-022-17583-4)
Supplement: Supplementary file 1 — Supplementary Figures. [file 41598_2022_17583_MOESM1_ESM.docx]

**SUPPLEMENTARY MATERIALS**


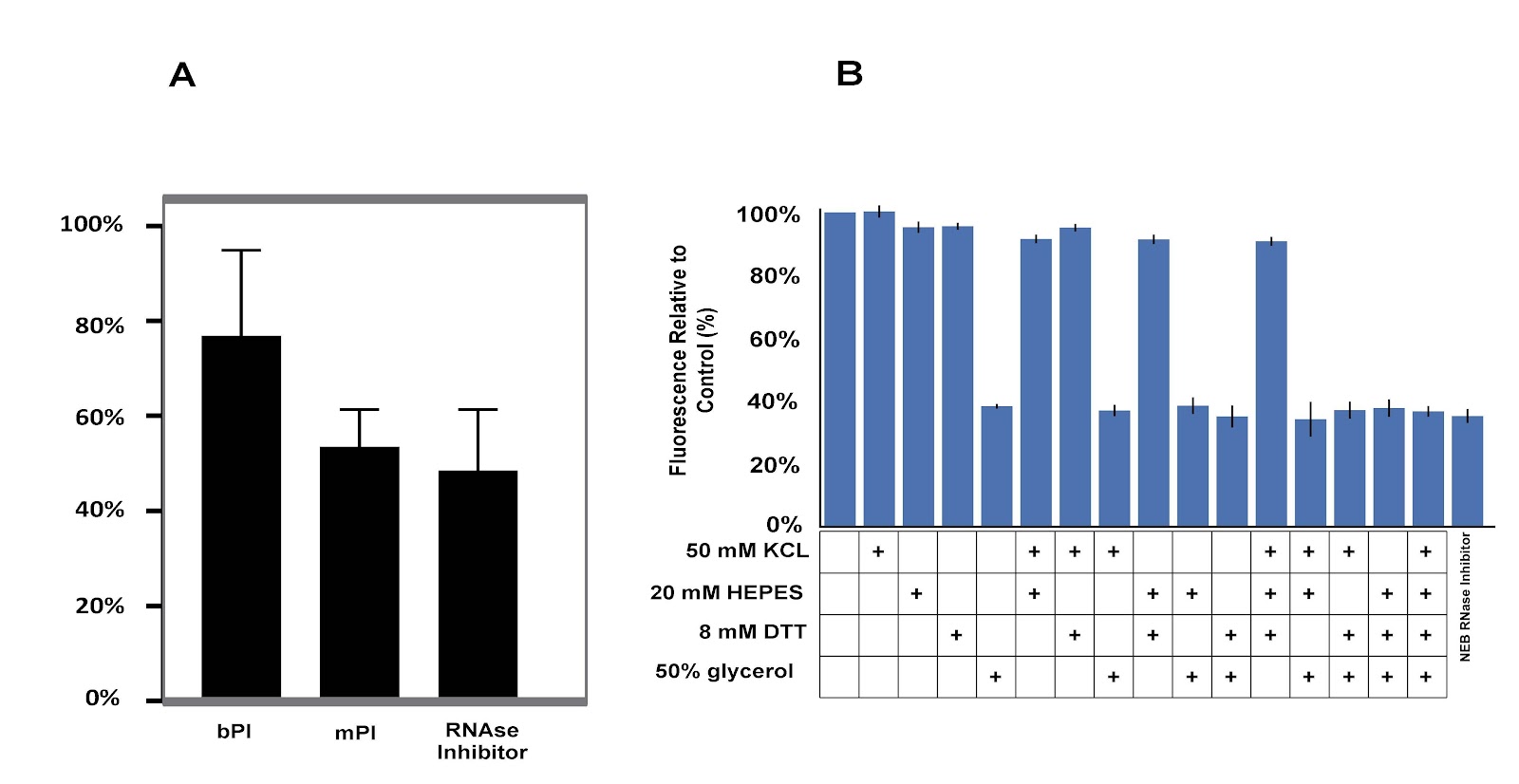


**Supplementary Figure 1**: Constitutive sGFP production in absence of clinical samples is diminished by the addition of commercial enzyme inhibitors (mammalian protease inhibitor, bacterial protease inhibitor, and RNase inhibitor). Cell-free reactions were run at 37°C in a sealed 384-well plate for 120 minutes in the presence or absence of RNAse inhibitor, mammalian protease inhibitor, or bacterial protease inhibitor. Data were normalized to a control with no inhibitor and are the mean of three experiments performed on three different days. Error bars correspond to ±SD.


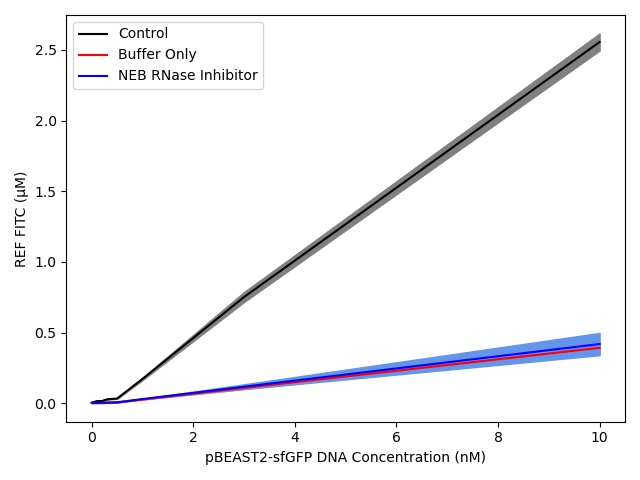


**Supplementary Figure 2**: sfGFP expression vs. constitutive DNA concentrations (0-10 nM) for the positive control (standard cell-free reaction mix) and in the presence of RNase inhibitor or equivalent buffer concentration without purified protein (50 mM KCl, 20 mM HEPES, 8 mM DTT, 50% glycerol). Data are the mean of three experiments performed on three different days. Shaded areas correspond to ±SD.


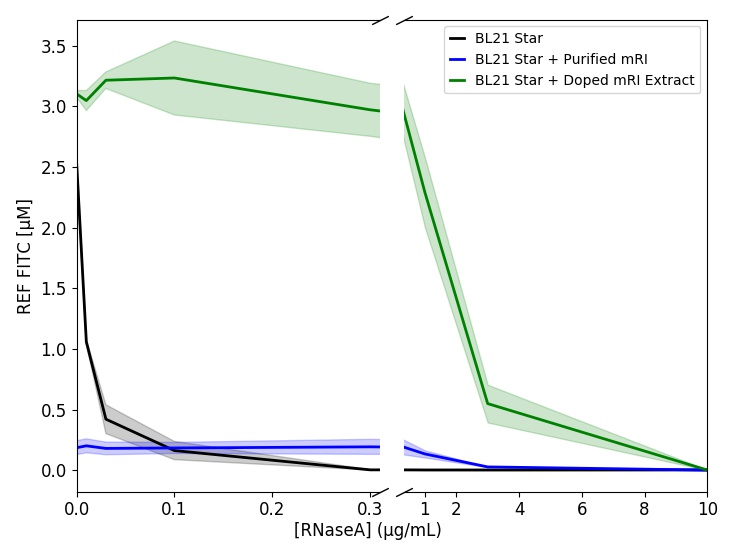


**Supplementary Figure 3**: Absolute sfGFP fluorescence (in FITC units) in standard extract (BL21 Star), standard extract with commercial RNase inhibitor (BL21 Star + Purified mRI), and extract induced to produce RNase inhibitor during cell growth (BL21 Star + Doped mRI Extract) when challenged with an RNaseA concentration gradient. Data are the mean of three experiments performed on three different days. Shaded areas correspond to ±SD.
